# Molecular Variability and Genetic Structure of *Chrysodeixis includens* (Lepidoptera: Noctuidae), an Important Soybean Defoliator in Brazil

**DOI:** 10.1371/journal.pone.0121260

**Published:** 2015-03-27

**Authors:** Janine Palma, Kevin Maebe, Jerson Vanderlei Carús Guedes, Guy Smagghe

**Affiliations:** 1 Laboratory of Integrated Pest Management, Department of Crop Protection, Federal University of Santa Maria, Santa Maria, Rio Grande do Sul, Brazil; 2 Laboratory of Agrozoology, Department of Crop Protection, Faculty of Bioscience Engineering, Ghent University, Ghent, Belgium; Federal University of Viçosa, BRAZIL

## Abstract

This study provides the first genetic characterization of the soybean looper, *Chrysodeixis includens* (Walker, 1857), an important defoliating pest species of soybean crops in Brazil. Population genetic variability and the genetic structure of *C*. *includens* populations were evaluated by using ISSR markers with samples from the major soybean producing regions in Brazil in the growing seasons 2011/2012. Seven different primers were applied for population characterization of the molecular variability and genetic structure of 8 soybean looper populations from 8 states of Brazil. The seven ISSR loci generated 247 bands in 246 individuals of *C*. *includens* sampled. The expected heterozygosity (*H*_E_) in the populations varied between 0.093 and 0.106, while the overall *H*_E_ was 0.099, indicating low genetic diversity. The analysis of molecular variance indicated that 98% of the variability was expressed among individuals within populations (*F*_ST_ = 0.021, *p* = 0.001). The low level of polymorphism over all populations, the high levels of gene flow, and the low genetic structure are indicatives of the exchange of genetic information between the different sampled regions. Population structuring suggests the presence of two major groups which do not correlate with their geographic sampling location in Brazil. These results may indicate recent recolonization of *C*. *includens* in Brazil or migration patterns following source-sink dynamics. Furthermore, the presence of two groups within *C*. *includens* suggests that a study on development of resistance or any other genetic-based trait needs to be evaluated on both groups, and pest management in soybean fields should be aware that differences may come to the control strategies they use.

## Introduction

The distribution of the soybean looper, *Chrysodeixis includens*, is restricted to the Western Hemisphere, from the USA to southern South America, and Australia [1]. The origin of this species is unclear. But in most parts of the USA, *C*. *includens* is known to migrate, with annual emigrations of moths to the northern states. Florida, Central and South America and the Caribbean islands are reservoirs for overwintering populations [2, 3]. The caterpillar has a broad host range, including 73 plants from 28 different families [1]. In Brazil, this species is considered to be a pest species of important summer crops like soybean, cotton, beans and tomato. Hence, the caterpillars are also found in crops of mild weather such as some Brassicaceae: e.g. broccoli, cabbage and watercress [4]. In the South of Brazil, *C*. *includens* was even found feeding on radish during the winter (own observations). The duration of the egg stage of *C*. *includens* range from 3 to 5 days. The species go through 5 to 6 instars in a period of 23.3 days. As the whole life cycle is 43 days at 27°C [5], it has the potential to generate 3 cycles in soybean culture.

*Chrysodeixis includens* has caused significant damage to soybean, especially in the producing regions of Brazil [6–8]. Until the 90s the soybean looper was considered to be a secondary pest with: a low distribution, isolated appearances, and small populations when present in the crop cycle [9]. Indeed, the importance increased due to surges of the caterpillar in several states over the years, occurring isolated or in association with another defoliating caterpillar, *Anticarsia gemmatalis*, in the soybean vegetative phase [7, 9]. The soybean looper has received more and close attention as a pest species in Brazil, due to a reinfestation event in soybean fields, with an increase in population numbers at the end of the culture cycle [10]. Before designing strategies for controlling this pest species in soybean, we need information about the population structure and gene flow of this species.

The control of the soybean looper in Brazil is currently in a transition period, between the use of insecticides and the use of genetic modified soybean that expresses the insecticidal protein Cry1Ac of *Bacillus thuringensis*, with currently no resistance observed [11]. However, in the USA high levels of resistance to pyrethroid insecticides has been reported in field populations [12, 13]. The determination of the population genetic structure, as well as the gene flow among different geographic areas have a practical application in pest control programs. As an example, they could help in designing appropriate resistant management strategies, preventing the rapid dispersion of resistance when it is detected in field populations [14, 15].

In general, DNA methodologies are contributing to our knowledge of genetic diversity and population structure [16]. Molecular markers are used to estimate gene flow and genetic variation [17]. Over the last decade several studies have attempted to determine the genetic characterization of populations from different species of the Noctuidae family using different molecular markers [18–23]. However, until now there was no study which molecularly characterized populations of the soybean looper.

Here in this study, we performed the first molecular characterization of *C*. *includens* with the use of Inter Simple Sequence Repeat (ISSR) markers. ISSR markers were widely used to characterize plants [24], and are more recently used also in insects to study the populations of Lepidoptera [25–28], Coleoptera [29], Diptera [30, 31], Hymenoptera [32], and Neuroptera [33]. ISSR markers are arbitrary markers produced by PCR, which permit the detection of polymorphisms in inter-microsatellite loci regions using specific primers designed from di-, tri- or tetra-nucleotide sequence repeats. ISSR markers have the advantage of: requiring no prior information of DNA sequence of the target species, and producing fragments with higher reproducibility, than RAPD (Randon Amplified Polymorphic) markers [31].

The aim of the present study was to detect patterns of genetic variation in populations of *C*. *includens*, and to get information about the population structure and gene flow of this pest species. This information could become essential in designing strategies for controlling this pest species in soybean, and provide more insights useful in the decision-making for resistance management.

## Materials and Methods

### Insect samples

Caterpillars were collected from several soybean production regions in Brazil (Table 1) (collection permit emitted by Brazilian Institute of Environment—IBAMA/SISBIO # 32627–2), and were kept in 96% ethanol for transport to the Integrated Pest Management Laboratory (LabMIP) in the Plant Health Protection Department of the Federal University of Santa Maria (UFSM) where they were stored and frozen in microcentrifuge tubes at -20°C. The immatures of *C*. *includens* were identified in LabMIP by morphologic characterization [34–36]. Each sampling location was considered as a separate population.

**Table 1:** Details of the eight populaions of *C*. *includens* included in this study.

**Code**	**State (cities)**	**Sample size**	**Latitude** ^**a**^ **(S)**	**Longitude** ^**a**^ **(O)**	**Collection date**
**TO**	Tocantins (Alvorada, Porto Nacional)	20	11°30'46"	48°44'45"	Jan/2012
**BA**	Bahia (Correntina, Barreiras, São Desidério, Formosa do Rio Preto)	30	12°24'35"	45°57'25"	Jan/2012
**MG**	Minas Gerais (Unaí, Paracatu, Coromandel), Goiás (Formosa)	30	16°48'10"	47°8'24"	Fev/2012
**GO**	Goiás (Americano do Brasil, Edéia, Rio Verde)	30	17°02'56"	50°16'54"	Jan/2012
**MT**	Mato Grosso (Itiquira, Rondonópolis), Mato Grosso do Sul (Chapadão do Sul)	20	17°40'37"	53°52'51"	Jan/2012
**MS**	Mato Grosso do Sul (Naviraí, Dourados, Maracajú)	30	22°17'31"	54°36'57"	Fev/2012
**PR**	Paraná (Tibagi, Londrina, Campo Mourão, Cruzeiro do Oeste)	41	23°56'09"	51°48'54"	Fev/2012
**RS**	Rio Grande do Sul (São Vicente do Sul, Frederico Westphalen, Palmeira das Missões, Santa Maria)	36	28°42'07"	53°46'44"	Fev/2012
**Total**		246			

^a^ Latitude and longitude are the average of decimal coordinates of each location.

### DNA extraction

The caterpillars were dissected, and the integument was used for DNA extraction. The cell lysis based protocol of Sarkosyl [20] was first tested (data not shown) and then performed with some small modifications. After the separation of the organic phase with chloroform/isoamyl alcohol, 10 μg mL^-1^ of RNAse was added, and then incubated at 37°C for 30 min. Then, the DNA was precipitated by the addition of 2 times the amount of the final volume of cold 100% isopropanol and 45% of the final volume of 5 M ammonium acetate. At the end of each extraction the pellet was resuspended in 25 μl of Milli-Q water, and stored at -20°C. A total of 50–200 ng of genomic DNA was observed by electrophoresis on 1% agarose gel using 1X TBE buffer (Tris/Acid Boric/EDTA) at 80 V.

### ISSR amplification

Molecular analysis was performed using seven ISSR primers (Table 2). The primers, which consist out of (CA)n + base, are repeated sequences that appear most frequently in the genome of Lepidoptera [25, 37]. The other primers were tested to different species of Noctuidae, Pyralidae, Sphingidae and Pieridae [25, 26].

**Table 2:** List of ISSR primers used for characterization of *C*. *includens*.

**Code**	**Sequence (5’-3’)**	**Annealing temp (°C)**	**Size range (pb)**	**Number of multiple bands**
**CI 01**	CACACACACACACACACACAA	56	260–4800	34
**CI 02**	CACACACACACACACACACAT	56	280–4200	32
**CI 03**	CACACACACACACACACACAG	57	210–3700	35
**CI 04**	ACACACACACACACACACACC	54	180–4000	42
**CI 05**	GACAGACAGACAGACA	54	230–2700	29
**CI 06**	GGATGGATGGATGGAT	55	180–4000	38
**CI 07**	CTCTCTCTCACACACACA	55	260–4200	37
**Total**				247

Two hundred forty six individuals were used for the ISSR polymerase chain reaction (PCR) according to Liu *et al*. (2006), with 1 μl genomic DNA, and 0.5 μM of primer. In the amplification reaction samples were initially denatured at 94°C for 2 min, followed by 37 cycles at 94°C for 30 s, annealing at their respective temperature for 45 s (Table 2), and extension at 72°C for 1.5 min. The PCR protocol ended with a final extension step at 72°C for 20 min [38]. A negative control PCR tube was run with each primer to check for contamination.

ISSR products were visualized by electrophoresis using 1.5% agarose gel at 100V for 40 min in 0.5X TAE buffer (Tris/Acetate/EDTA). After staining with ethidium bromide buffer, the banding patterns were visualized under U.V.-light and photographed. The Mass Ruler DNA Ladder Mix molecular weight marker (Thermo Scientific, Pittsburg PA, USA) was ran as a standard with each primer. To ensure PCR quality, we repeated the PCR for all primers on some samples. Each primer was capable of producing a reproducible banding pattern.

### Data analysis

The ISSR band profiles from each DNA sample were analyzed using the Bionumerics software (Applied Maths, Belgium), and scored for the presence (1) or absence (0) of bands for each primer, excluding weak and/or smeared bands. Thus, a binary matrix (1/0) was generated, with 247 multiple bands from 246 individuals.

Genetic diversity for each population was estimated through the percentage of polymorphic loci (P), the mean effective number of alleles (*N*e), the mean expected heterozygosity (*H*_E_), and the mean Shannon’s Information Index (*I*), assuming Hardy-Weinberg Equilibrium by using the GenAlEx 6.5 software [39, 40].

Analysis of Molecular Variance (AMOVA) was conducted to calculate the variance among and within populations [41]. Genetic differentiation coefficients between populations were calculated as *F*_ST_ by using the GenAlEx 6.5 software [39, 40]. The significance was established by 999 permutations. Gene flow (*N*m) was calculated as *N*m = (1-*F*_ST_)/2*F*_ST_ [42].

The population structure of *C*. *includens* was determined by performing a Mantel test, Principal Coordinate Analysis (PCA) and Bayesian approach. The Mantel test, which assumes a linear correlation between geographic distance and genetic distance matrices, was done by using the GenAlEx 6.5 software with 9,999 permutations [39, 40]. The PCA was executed with the pairwise genetic distance matrix in GenAlEx 6.5, while the Bayesian approach was performed by using the STRUCTURE v. 2.3.3 software [43]. The latter approach assumes a model with admixture of populations, starting from a single random-mating population and determines the number of populations that are more appropriate for interpreting the data. The number of populations (K) was estimated from 1 to 10 for each value with 250,000 burn-in iterations and 750,000 data collecting steps and were repeated 9 times. The ΔK of the Evanno method [44] was performed to estimate the value of K that best fitted the data, by using the Structure Harvester v. 0.6.93 software [45]. The graphical visualization of the population structure was produced with the Distruct v. 1.1 software [46].

## Results

### Genetic variability

The seven ISSR primers provided clear, consistent and reproducible band patterns. With these primers, we scored a total of 247 ISSR bands, varying from 180 to 4800 pb, for 246 individuals of *C*. *includens* sampled from eight populations formed from sample locations grouping. All loci were 100% polymorphic. The ISSR primer which amplified the highest number of bands was CI04 with 42 bands, and the lowest number of bands was, with 29, produced by primer CI05 (mean number of bands = 35.28) (Table 2).

The population with the highest percentage of polymorphism (P) was PR (86.20%), while the lowest percentage occurred in TO and MT (68.80%) with a mean within population polymorphism rate of 77.48%. The overall populations mean expected heterozygosity (*H*_E_) and Shannon’s information index (*I*), both parameters of genetic diversity, were 0.099 and 0.184, respectively. The population PR showed the highest genetic diversity with *H*_E_ = 0.106 and *I* = 0.200. While MT showed the lowest genetic diversity with *H*_E_ = 0.093 and *I* = 0.173. The mean effective number of alleles (*N*e) within the populations was 1.124 (Table 3).

**Table 3:** Genetic diversity of different *C*. *includens* sampling sites.

	**P** ^**a**^ **(%)**	***N*e** ^**b**^	***I*** ^**c**^	***H***_**E**_ ^**d**^
**TO**	68,8	1,131	0,182	0,100
**BA**	81,7	1,116	0,178	0,094
**MG**	78,1	1,126	0,185	0,100
**GO**	81,3	1,132	0,196	0,105
**MT**	68,8	1,118	0,173	0,093
**MS**	76,1	1,119	0,177	0,095
**PR**	86,2	1,131	0,200	0,106
**RS**	78,5	1,123	0,183	0,098
**Mean**	77,48	1,124	0,184	0,099

^a^ *P* = percentage of polymorphic loci;

^b^ *N*e = mean effective number of alleles;

^c^ *I* = mean Shannon’s information index;

^d^ *H*_E_ = mean expected heterozygosity.

### Population genetic structure

The analysis of the genetic structure of the *C*. *includens* populations (AMOVA) indicated a low but significant genetic differentiation among populations (*F*_ST_ = 0.021; *p* = 0.001), with 98% of the overall genetic variability occurring within populations and only 2% of the variation occurring among populations (Table 4). Furthermore, the Mantel test detected structuring with a low but not significant correlation between genetic and geographic distances (r = 0.192; *p* = 0.210). The estimated amount of gene flow between the populations, indirectly calculated from *F*_ST_, was high (*N*m = 23.197), indicating high migration among sampling localities.

**Table 4:** Analysis Summary of molecular variance (AMOVA) based on sampling sites of *C*. *includens*.

**Sorce of variation**	**D.F.**	**Sum of Squares (SS)**	**Variance components**	**% of variation**
**Among populations**	7	247.099	0.459	2
**Within populations**	238	5069.044	21.299	98
**Total**	245	5316.142	21.758	100

*p* < 0.0001

**Fig 1:**
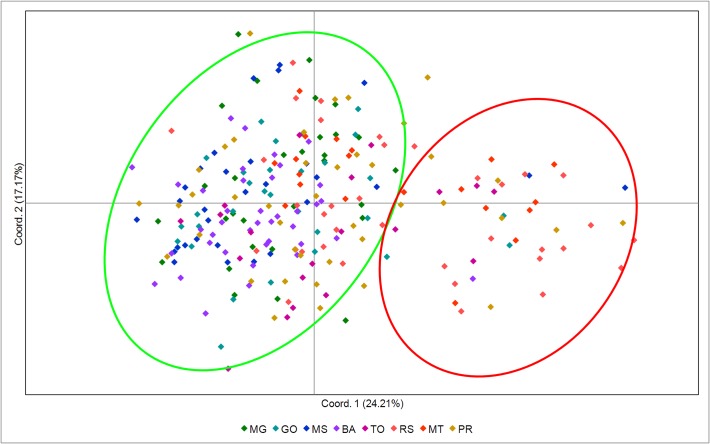
Principal coordinate analysis (PCA) of *C*. *includens*. The codes used here, correspond to the different populations described in Table 1. The ellipses correspond to the two groups formed.

The PCA of all individuals from the eight populations revealed two groups. The first Principal Component (PC1, Component X) explained with 24.21% most part of the variance, while the second Principal Component (PC2, component Y) explained 17.17% of the total variance. No clear geographical pattern between both groups was observed (Fig 1).

**Fig 2:**
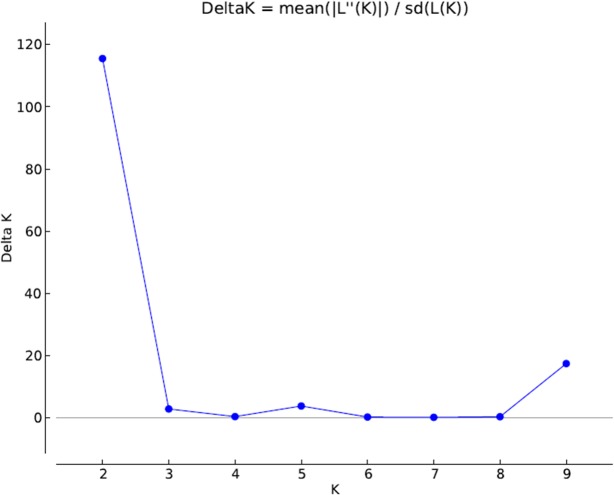
ΔK calculated by the Evanno method. The test was performed with all *C*. *includens* individuals from K = 1 to K = 10.

**Fig 3:**
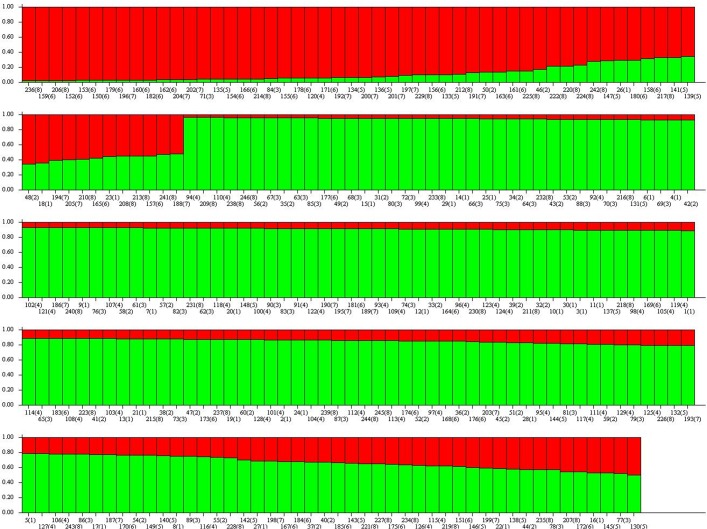
Cluster analysis using STRUCTURE software (K = 2). Different colors represent different genetic groups. Each column represents an individual. The numbers 1 to 8 correspond to codes MG, GO, MS, BA, TO, RS, MT, and PR, respectively.

**Fig 4:**
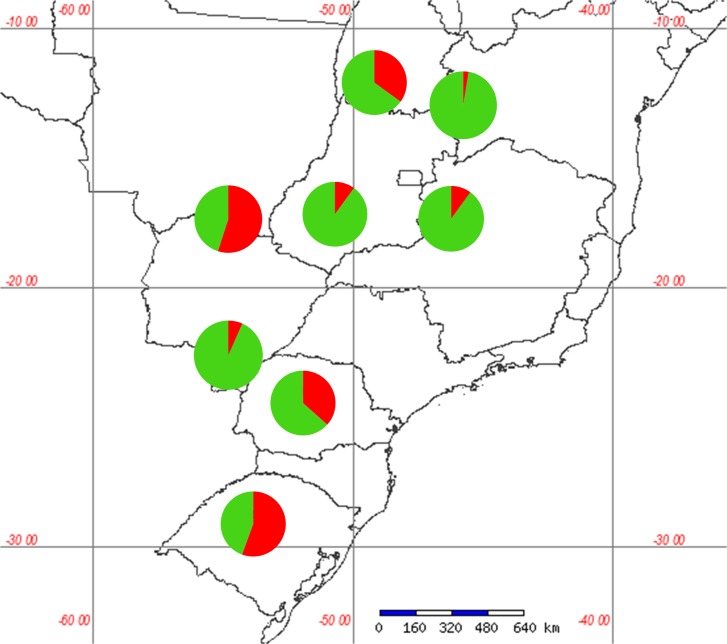
Distribution map of two *C*. *includens* groups identified with PCA and Structure. Pie charts are arranged on the geographical position of each sampled population. Each pie chart indicates the proportion of each group in the populations. Codes correspond to the populations described in Table 1.

The cluster analysis suggested two different genetic groups. Indeed, the Evanno method identified ΔK = 2, this is the best value of K (or number of populations) that fitted our data (Fig 2). Each specimen was assigned to one of this two groups (Fig 3). The suggestion of those two groups was similar to the division obtained by the PCA, with no indication of structuring of the samples by location (Fig 4). Furthermore, the clusters in Fig 4 suggest an exchange of genetic information between both groups.

## Discussion

Here in this study, we performed the first molecular characterization of *C*. *includens* with the use of ISSR markers. To determine the genetic characterization of the soybean looper in Brazil, we conducted an analysis of the genetic diversity and population structure across eight populations, utilizing seven ISSR molecular markers. We have chosen in this project to work with ISSR markers because they allowed us to detect a high level of polymorphic bands (100%). This is comparable with the polymorphism levels detected with ISSR markers in other species. For example, in *Lymantria dispar*, *Ceratitis capitata* and *Plutella xylostela*, the polymorphism levels were 100%, 85% and 100%, respectively [27, 31, 47]. These high levels could be due to the high mutation rate, and/or insertion or deletion events within the microsatellite sequences and/or within the amplified region [48]. In general, the latter studies have proven that ISSR markers were efficient in the detection of the genetic variability in the studied populations.

In our study, the gene flow over all populations, estimated from the *F*_ST_ value, was high (23.197). The Mantel test detected a low but not significant correlation between genetic and geographic distances. Despite this low genetic differentiation between the *C*. *includens* populations, the PCA and Bayesian approach test divided all individuals in two major groups which were not correlated with the geographical origin of the specimens. The high number of migrants among the populations of *C*. *includens* could be responsible for the low differentiation observed here. Although there is currently no information available on the direction and the migration rate of *C*. *includens* in Brazil, the soybean planting pattern together with the polyphagous character of species agrees with facultative migratory habits [2, 4]. Furthermore, in most parts of the USA, *C*. *includens* is known to migrate [3].

The high level of gene flow of *C*. *includens* in Brazil also suggests a movement of the species between host plants along the cultivated areas in Brazil. Soybean plantations in Brazil start in the Mato Grosso state and progress to other states in the Southeast, Midwest and Northeast following the onset of the rain season, and the increase of the temperature in the Southern regions. These temporal climatic changes could encourage the migration of moths to new areas with soybean, considering that moths of *C*. *includens* have preference to oviposit on soybean leaves [49]. Indeed, it is known that caterpillars have a food preference for soybean plants [50], and young larvae prefer to feed on young plants with low-fiber-containing leaf tissues of high digestibility [51]. However, this result cannot determine if the migration occurs each season and/or how they migrate overtime within one season. Although these results indicate migration between distant populations, remnant soybean plants and other host plants can also function as reservoir or source population of soybean loopers during and after the winter [52]. In this context, polyphagous pest species such as the soybean looper, could show also resident individuals, once oviposition sites and food sources are available [21].

The results of this study may also be indicative of a recent recolonization of *C*. *includens* in Brazil. Indeed, the soybean looper is able to migrate long distances as (i) is seen in the USA where *C*. *includens* is recognized as a migration species, (ii) by the absence of overwintering and recolonization of *C*. *includens* in some states [51], and (iii) the permanent presence of *C*. *includens* in Florida where it can survive at average temperatures of 16°C [2]. The states of Florida and Texas as well as the Caribbean islands, and Central and South America are considered to harbor the source population of *C*. *includens* [3]. Therefore, the origin of *C*. *includens* in Brazil may be the Caribbean islands or Central America. However, to investigate the origin of Brazilian *C*. *includens* populations, a new analysis including samples from different Central and North American countries, and the Caribbean islands would be necessary.

In this study we estimated that the gene flow over all populations was high (23.197). Values greater than nine migrants per generation were sufficient to homogenize populations [53]. Although natural selection tends to adapt populations to local environmental conditions, immigrants from other populations will introduce genes adapted to other conditions and will homogenize these populations [17]. Homogenous populations under high insecticide pressure, as occurs in Brazil, may exhibit insecticide resistance development under high level of gene flow, even when the initial frequency of resistant allele is low [54]. In Brazil, insecticide resistance is not yet reported but in the USA high levels of resistance against pyrethroids are known since 1990 [13]. However, year by year the soybean looper is more difficult to control in Brazil, in part due to the indiscriminate use of insecticides. The use of insecticides that kill both pest species and their natural enemies could lead to an increased number of surviving pest species. Indeed, due to population dynamics and a high activity of detoxifying enzymes, polyphagous pest species often develop resistance before their parasites or predators do [55]. As the population of natural enemies will decrease faster, they will lose their biological control potential. And thus in time, the pest density will depend more on insecticide use, which in turn will increase the resistance evolution [55].

In conclusion, we present here the first molecular study on the populations of the soybean looper, *C*. *includens*. Our results showed high levels of gene flow and low genetic differentiation between the different populations, which may indicate recent recolonization of *C*. *includens* in Brazil and/or migration patterns following source-sink dynamics. Further research is needed to identify the migration patterns of *C*. *includens* in Brazil. Furthermore, the presence of two groups within *C*. *includens* suggests that any management tactic dependent on insect genetics such as the effect of insecticides and the development of resistance, needs to be evaluated on both groups. Taking this into consideration, pest management in soybean fields should be aware that differences may come to the control strategies they use.
